# Commentary on the role of treatment-related HIV compensatory mutations on increasing virulence: new discoveries twenty years since the clinical testing of protease inhibitors to block HIV-1 replication

**DOI:** 10.1186/1741-7015-10-114

**Published:** 2012-10-03

**Authors:** Eric J Arts

**Affiliations:** 1 Division of Infectious Diseases and HIV Medicine, Department of Medicine, School of Medicine, Case Western Reserve University, Cleveland, Ohio, USA; 2 Department of Molecular Biology and Microbiology, School of Medicine, Case Western Reserve University, Cleveland, Ohio, USA

**Keywords:** Antiretroviral drugs, drug resistance, CD4 cell counts, fitness, human immunodeficiency virus, protease inhibitors, viral load, virulence

## Abstract

Approximately 20 years has passed since the first human trial with HIV-1 protease inhibitors. Protease inhibitors set the stage for combination therapy in the mid-1990s but are now rarely used in first-line combination therapy and reserved for salvage therapy. Initially, resistance to protease inhibitors was deemed unlikely due to the small enzymatic target with limited genetic diversity, the extended drug binding site in protease, and the need to cleave multiple sites in the HIV-1 precursor proteins. However, a highly protease inhibitor-resistant virus can emerge during treatment and is found to harbor a collection of primary drug-resistant mutations near the drug and/or substrate binding site as well as secondary mutations that compensate for fitness loss. For years, the research field has debated the impact of these secondary mutations on the emergence rates of high-level protease inhibitor resistance. A recent study poses a more pertinent question, related to disease progression in patients newly infected with a virus harboring secondary protease inhibitor-associated polymorphisms. The authors of that study show that increased rates of disease progression, inferred by increased viral loads and decreased CD4 cell counts, correlate with a fitness score of the infecting virus. The modeled fitness scores increased with an accumulation of these secondary protease inhibitors mutations, and not because of any one specific polymorphism.

## Background

In 1992, I attended a Gordon Conference entitled the 'Chemotherapy of AIDS' in Oxnard, California. My notes on this conference, the program, and the collection of abstracts are buried in some dark and scary corner in my office. Fortunately, this meeting has been etched in my memory for the past 20 years, and not just for the good food and inebriating beverages. Nucleoside RT inhibitors (NRTIs) like zidovudine, didanosine, and even zalcitabine (also known as ddC for those who forgot) were still being tested in various dual combination therapies with three to six months of sustained virus control [1,2]. Resistance was rampant with even dual NRTI therapy and yet, most pharmaceutical companies still held out a glimmer of hope that their next antiretroviral drug would work in monotherapy. At this meeting, all the pharma giants rolled out presentations on their protease inhibitors (PIs) that were being tested in phase I or II clinical trials. I remember a gaggle of chemists in the room begging presenters to keep their slides up so they could quickly scratch down the various modifications on these peptidomimetic inhibitors. Some of these PIs maintained 10-fold drops! in HIV-1 levels for up to six months without the emergence of resistance [3,4]. At the time, the other new drugs on the block, lamivudine and nevirapine, were deemed colossal failures because viral load rebounded with a resistant HIV-1 variant almost immediately with monotherapy [5,6]. Oh, how times have changed. A triple drug combination therapy, based on a non-NRTI (NNRTI)backbone, is now the norm for first-line therapy and anything less than complete virus suppression is deemed a treatment failure (reviewed in [7]).

**Figure 1:**
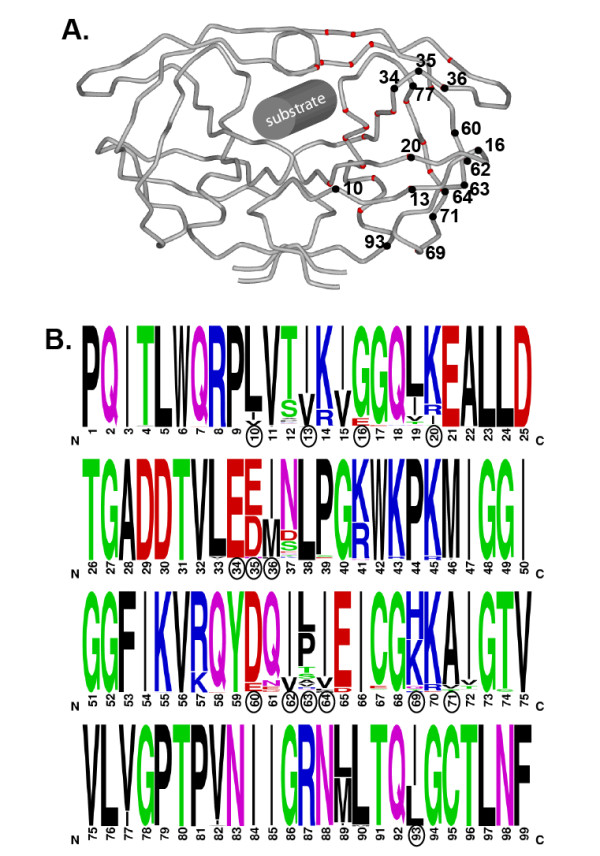
**Schematic representation of the HIV-1 protease wireframe crystal structure and highlighting the secondary/compensatory selected under protease inhibitor treatment**. **(A) **The wireframe cartoon of the protease X-ray crystal structure (PDB ID:3HVP ) is present to highlight the position of the secondary mutations (black dots) that compensate for fitness losses derived from primary drug resistance to protease inhibitors (red dots). **(B) **A schematic representation of the genetic diversity in the HIV-1 protease derived from the curated HIV-1 protease alignment from Los Alamos National Laboratories HIV sequence database. Alignments of protease and any amino acid found at >1% frequency is presented in this WebLogo (http://weblogo.berkeley.edu).

Aside from their antiviral potency on HIV in tissue culture, PIs were perceived as better than the rest because of their small enzymatic target with a pleotropic cleavage pattern. On average, 50 HIV-1 proteases, comprising a 198-amino acid homodimer (Figure 1A), must cleave approximately 2,000 to 4,000 Gag and Gag-Pol proteins at eight sites to obtain an infectious virus particle. With such high binding affinity and selectivity of PIs for HIV-1 protease, it appeared inconceivable that this enzyme could accommodate resistance and maintain cleavage of the natural substrates. However, the vast combination of protease mutations associated with resistance swept away our preconceived notions of HIV-1 drug resistance. One unique feature of the mutational pattern leading to PI resistance, which had not been readily observed in viruses with resistance to NRTI or NNRTI, was the accumulation of mutations outside of the substrate or inhibitor binding site [7]. As outlined in Figure 1A, primary drug-resistance mutations are positioned below the 'flap' and near the substrate or inhibitor binding groove (red dots in the wire frame protease structure; Figure 1A) whereas secondary mutations (for example, L10I/V, I13V, K20I/M/R, M36I, D60E, I62V, L63P, A71T/V, V77I and I93L) are present primarily on solvent accessible regions near the surface of the dimer. The effects of these secondary PI mutations has been a subject of debate for years but a recent paper by Theys *et al*. in *Retrovirology *[8] sheds new light on how these polymorphisms may impact disease progression in newly infected patients.

## Discussion

How do these mutations emerge under PI selection? Most primary PI-resistance mutations may pre-exist at low frequency in the intrapatient HIV-1 population but their dominance with PI treatment or selection comes with a significant fitness cost [9]. Subsequent emergence of secondary mutations is thought to compensate or dampen this fitness loss [10]. An alternative hypothesis suggests that these 'secondary' mutations actually emerge first under drug selection to compensate and provide a framework for the emergence of primary drug-resistance mutations [11]. Regardless, the pathway to PI resistance under PI treatment is extremely complex and likely affected by both the baseline protease sequence of the HIV-1 population and additional selective pressures, for example, overlapping cytotoxic T cell epitopes and maintaining recognition of eight cleavage sites. As described in Figure 1B and based on the Los Alamos HIV-1 sequence database, we now know that most of the 99 amino acid sites are tolerant to some sequence change in the presence or absence of PI treatment.

Initially, compensatory mutations were poorly distinguished from primary mutations conferring PI resistance. A plethora of research articles identified PI treatment-associated 'mutations' in treatment-naïve populations, especially in those infected with non-subtype B HIV-1 [7]. Thus, the impact of these secondary mutations (which could also be wild-type sequence for some HIV-1 subtypes) on treatment outcome and emergence of PI resistance was immediately brought into question and still without resolution to date. However, a more important role for these secondary mutations may relate to replicative fitness and the ability to compensate for the fitness loss associated with primary PI-resistance mutations. In absence of the primary PI-resistance mutations, these secondary mutations or polymorphism may actually increase fitness and, as a consequence, virulence. This must be qualified with a big IF because the hypothesis assumes that replicative fitness is a direct and possibly dominant correlate of disease progression. I have spent the last 15 years of my research career providing evidence to support this virulence hypothesis [12,13] so the excellent article by Theys *et al*. [8] serves my ego well.

**Figure 2:**
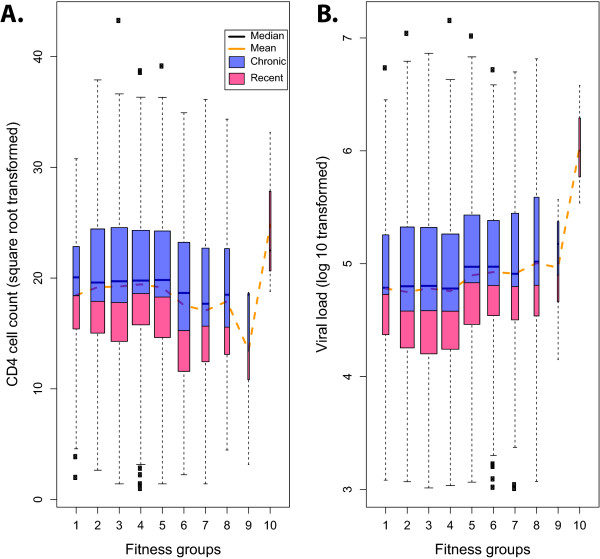
**Relationship between HIV-1 fitness with protease inhibitor-associated compensatory mutations and (A) CD4 cell counts or (B) viral loads**. For a population of recently diagnosed drug-naïve patients, a fitness landscape was used to estimate *in vivo *HIV fitness under protease inhibitor drug selective pressure. Estimated viral fitness was discretized into 10 groups, and the distribution of viral load and CD4 cell count was plotted using boxplots. The widths of boxplot are proportional to the number of samples used. The proportion of patients with indications of recent (acute) infection within each fitness group is shown in red.

Establishing fitness scores from the relative proportions of HIV-1 genotypes in the human population requires the development of a robust evolutionary framework. In a previous study [14], the frequencies of synergistic interactions between mutational pairs in protease were compared with the frequency of any one mutation alone, that is, the principle behind epistasis in fitness. With protease sequences from over 12,000 treatment-naïve and PI-treated patients, a fitness score could be estimated for specific PR genotypes based on their prevalence in the subtype B epidemic. A similar approach was used to score fitness of reverse transcriptase genotypes. Higher fitness scores were observed in patient virus with an accumulation of these secondary mutations rather than any single protease polymorphism. Higher fitness scores were also a significant correlate of lower CD4 cell counts and higher viral loads (Figure 2 provided by Theys *et al*.). Reverse transcriptase polymorphism showed no association with these clinical parameters.

The outcome of disease following transmission of HIV-1 with primary drug-resistant mutations is still a subject of great debate. In the study by Theys *et al*. [8], lower fitness scores derived from primary drug-resistance mutations did not correlate with higher CD4 cell counts or lower viral loads in treatment-naïve patients. The fitness cost of deleterious, escape mutations within a virus appears highly dependent on the time of selection pressure, relative appearance of the escape, and the rate of subsequent diversifying evolution that can lead to compensatory mutations. As we observed with emergence of cytotoxic T lymphocyte-escape mutations [15], compensation mutations rapidly emerge in HIV with a resumption of high virus turnover and with the intrinsically high mutation rates. Thus, some transmitted HIV-1 with primary drug resistance may be more fit than others and may not impact normal disease progression (in the absence of treatment). Likewise, reversion of the primary drug-resistance mutations in a background with compensatory mutations may result in HIV-1 that is actually more fit than the majority of wild-type virus: the central thesis of Theys *et al*. [8] If this fitness is related to virulence, infection with HIV-1 harboring PI-associated compensatory mutations may actually lead to faster disease progression as described (Figure 2) [8]. The disconcerting aspect of this hypothesis is the probable maintenance of this virulent HIV-1 throughout disease whereas deleterious mutations in a defective HIV are more likely to revert after transmission. There is, however, a fundamental concern that questions the foundation of the Theys *et al*. model. Why are these compensatory polymorphisms not found at even higher frequencies in the treatment-naïve HIV-1 population if they increase fitness? It is possible that the presence of compensatory mutations requires a specific evolutionary pathway through the fitness landscape. Alone, each of these polymorphisms may result in a slight fitness cost but in order to survive, the virus must amass these small 'fitness hits' to form a genotype that might compensate for the high fitness cost of the primary drug-resistant mutation. In this example, appearance of this virus with higher virulence is unlikely without the PI selective pressure directing the evolutionary pathway.

## Conclusions

Theys *et al*. [8] may have provided the first *in vivo *evidence that some HIV-1 mutants selected under drug pressure may actually be of higher replicative fitness, which in turn could lead to higher viral loads and lower CD4 cell counts. Furthermore, it is naturally assumed that all evolutionary pathways leading to drug resistance come with a fitness cost, but this is not the case. The fitness cost of NNRTI resistance is minimal at most [16] and multiple HIV-1 lineages evolved with intrinsic NNRTI resistance, such that mutation(s) to drug-sensitive strains actually results in reduced replicative fitness [17]. In addition, several HIV-1 isolates resistant to entry inhibitors, such as C-C chemokine receptor type 5 antagonist, actually have higher replicative fitness than the parental wild-type strains [18]. Collectively, these observations emphasize the need for an immediate switch in treatment regimens upon the identification of drug resistance to avoid the emergence of compensatory mutations.

## Abbreviations

HIV-1: human immunodeficiency virus type 1; NNRTI: non-nucleoside reverse transcriptase inhibitors; NRTI: nucleoside reverse transcriptase inhibitors; PI: protease inhibitors; RT: reverse transcriptase.

## Competing interests

The author declares that they have no competing interests.

## Author's information

Dr Eric J Arts is a Professor of Medicine in the Division of Infectious Diseases and HIV Medicine at Case Western Reserve University (CWRU) in Cleveland, Ohio. He also serves as Director of the CWRU Center for AIDS Research Laboratories in Kampala, Uganda. His research focusses primarily on HIV-1 evolution, pathogenesis and drug resistance.

## Pre-publication history

The pre-publication history for this paper can be accessed here:

http://www.biomedcentral.com/1741-7015/10/114/prepub
